# Successful Case of Ovariotomy

**Published:** 1851-10

**Authors:** John Beale


*Successful Case of Ovariotomy.* By JOHN BEALE, ESQ.—The patient was a woman, aged 30, unmarried. The ovarian tumor was hard to the touch in the left iliac region and left hypochondrium, but soft and fluctuating on the opposite side, evidently in two distinct sacs, moveable, and not tender. It was removed on December 4, 1850, about a year after it was first perceived. The incision was ten inches in length, extending from the scrobiculus to the pubes. Two cysts were punctured, and their contents removed, before the tumor could be extracted; the pedicle was tied by a double ligature passed through its base, and the tumor was then separated as near as possible to it. The uterus and right ovary were healthy. Everything went on favorably; on the 15th of December, she was able to walk about the room; and on the 25th, the ligature came away. The tumor was 3 ft. 2 in. in its largest circumference, and 2 ft. 1½ in. in its smallest; it weighed 25 pounds; it was multilocular, marked on the surface by bands of white fibrous tissue corresponding with the septa of the cysts. The cysts varied very much in size, and in the density and tenacity of their contents; in the smaller ones, the fluid was clearer and thinner. The average specific gravity was 1010, but the fluid contained a very large quantity of albumen. The total amount of fluid was from 21 to 23 pints.—*Ibid.*

